# Reparo cirúrgico da avulsão tendínea proximal dos isquiotibiais

**DOI:** 10.1055/s-0045-1810039

**Published:** 2025-09-08

**Authors:** Guilherme Guadagnini Falotico, Bruno Francesco Scatigna

**Affiliations:** 1 Grupo do Quadril, Departamento de Ortopedia e Traumatologia, Escola Paulista de Medicina, Universidade Federal de São Paulo, São Paulo, SP, Brasil

**Keywords:** lesões dos tendões, músculos isquiossurais, tendões dos músculos isquiotibiais, hamstring muscle, hamstring tendons, tendon injury

## Abstract

As lesões do complexo dos músculos isquiotibiais proximais são frequentes em atletas e variam de distensões a avulsões tendíneas e ósseas. O mecanismo de lesão geralmente envolve contração excêntrica dos isquiotibiais durante hiperflexão abrupta do quadril com o joelho estendido. Lesões de baixa velocidade ocorrem em chutes altos e espacates, ao passo que avulsões tendíneas são comuns em atividades de alta velocidade, como corrida e balé. Clinicamente, os pacientes apresentam dor, hematoma subcutâneo e, em alguns casos, defeito palpável. Sinais adicionais incluem limitação na extensão do joelho e comprometimento do nervo ciático. O diagnóstico é feito por ultrassonografia, ressonância magnética (RM) e radiografia, sendo a RM o exame padrão. O tratamento cirúrgico é indicado para avulsões completas, especialmente em atletas, e visa evitar perda de força e dificuldades no retorno ao esporte. A técnica cirúrgica aqui descrita utiliza uma ou duas incisões transversais na prega glútea, dependendo da retração do tendão, com fixação por âncoras metálicas. O pós-operatório inclui restrição inicial, seguida de reabilitação acelerada para retorno ao esporte a partir da décima segunda semana. Desde 2019, a técnica foi aplicada em 13 pacientes, e demonstrou bons resultados sem rerrupturas, com pontuação pós-operatória no escore de Tegner semelhante à do pré-operatório.

## Abstract (en)

Injuries to the proximal hamstring muscle complex are common in athletes and range from strains to tendinous and bony avulsions. The lesion mechanism typically involves an eccentric contraction of the hamstring muscles during abrupt hip hyperflexion with the knee in extension. Low-speed injuries occur in high kicks and splits, whereas tendon avulsions are common in high-speed activities, such as running and ballet. Clinically, patients present with pain, subcutaneous hematoma, and, sometimes, a palpable defect. Additional signs include limited knee extension and involvement of the sciatic nerve. Diagnosis relies on ultrasonography, magnetic resonance imaging (MRI), and radiography, and MRI is the standard test. Surgical treatment is indicated for complete avulsions, especially in athletes, to prevent loss of strength and difficulty in returning to sports. In the surgical technique herein described, we perform one or two transverse incisions in the gluteal fold, depending on the tendon retraction, followed by fixation with metal anchors. The postoperative period includes initial restriction, followed by accelerated rehabilitation for return to sports by the twelfth week. Since 2019, the technique has been applied to 13 patients, demonstrating good outcomes, without re-ruptures and a postoperative Tegner score similar to the preoperative one.

## Introdução

As lesões do complexo dos músculos isquiotibiais proximais representam grande desafio para indivíduos fisicamente ativos e atletas competitivos. A gravidade dessas lesões pode variar desde distensões até rupturas completas miotendíneas, avulsões do tendão proximal dos isquiotibiais e avulsões ósseas. 1 2 O mecanismo de lesão geralmente envolve a contração excêntrica dos isquiotibiais secundária à hiperflexão abrupta do quadril, enquanto o joelho está estendido. 3 4 A ruptura miotendínea proximal dos isquiotibiais costuma ocorrer em lesões de baixa velocidade, como durante chutes altos, espacates e carrinhos deslizantes. 3 Por outro lado, as avulsões do tendão proximal dos isquiotibiais tendem a ocorrer em situações de alta velocidade, como corrida, esqui aquático ou posições extremas de amplitude no balé. 4

Clinicamente, os pacientes apresentam dor, hematoma subcutâneo e, em alguns casos, um defeito palpável ao longo do trajeto dos isquiotibiais. 5 Outros sinais incluem dor para s extensão do joelho na posição sentada, ausência de tensão nos músculos isquiotibiais (sinal do arco da corda) e comprometimento por contiguidade do nervo ciático, que pode cursar com déficits motores e/ou sensitivos, além de dor neuropática. 6 Para a confirmação do diagnóstico, diversas modalidades de imagem podem ser utilizadas, incluindo ultrassonografia, ressonância magnética (RM) e radiografia convencional, para avaliar o envolvimento ósseo, sendo a RM o método mais comumente utilizado. 7

As avulsões tendíneas completas e as avulsões ósseas representam possível indicação de tratamento cirúrgico, especialmente nos atletas competitivos, a fim de evitar longos períodos de recuperação que poderiam comprometer suas carreiras esportivas. O tratamento não cirúrgico dessas lesões está associado a índices de satisfação menores, redução da força dos músculos isquiotibiais e menor probabilidade de retorno ao nível esportivo pré-lesão. Quando a abordagem cirúrgica é realizada, os cuidados pós-operatórios devem priorizar a proteção inicial do reparo, seguida de um protocolo de reabilitação acelerado, que visa o retorno mais precoce possível ao esporte, após pelo menos 12 semanas da cirurgia. 8 9

O objetivo deste estudo é descrever a técnica de reparo aberto para avulsões tendíneas completas mediante uma incisão transversal na prega glútea para lesões com retração de até 5 cm e por meio de 2 incisões transversais para casos com migração distal do coto tendíneo maior do que 5 cm. O tratamento cirúrgico ainda é muito pouco debatido na literatura nacional, e não há técnica que seja habitualmente realizada pelos ortopedistas brasileiros.

## Descrição da Técnica

A técnica se baseia na experiência com uma série de 13 pacientes consecutivos, operados pelos mesmos cirurgiões em conjunto (GGF e BFS). A Tabela 1 evidencia os dados epidemiológicos, a classificação da lesão e os tempos de lesão e de seguimento.

**Tabela 1:** Dados epidemiológicos, classificação da lesão e os tempos de lesão e de seguimento

Sexo	Idade (anos)	Tempo de lesão	Esporte	Classificação	Tegner pré-operatório	Tegner pós-operatório	Tempo de seguimento	Complicações
Feminino	27	11 semanas	Corrida	3	7	7	42 meses	Não houve
Masculino	22	3 semanas	Rúgbi	2C	10	10	33 meses	Não houve
Masculino	46	7 semanas	Musculação	3	4	4	46 meses	Não houve
Masculino	48	2 semanas	Corrida	3	5	6	6 meses	Não houve
Masculino	53	2 dias	Esqui aquático	3	6	6	22 meses	Não houve
Feminino	16	4 semanas	Judô	2C	9	7	14 meses	Granuloma no fio de sutura, com reabordagem cirúrgica
Feminino	43	6 semanas	Musculação	2C	5	Não disponível	1 mês	Não houve complicações agudas
Masculino	52	2 semanas	Futebol	2C	5	Não disponível	1 mês	Não houve complicações agudas
Feminino	49	10 dias	Ciclismo e musculação	3	6	6	60 meses	Não houve
Masculino	34	12 semanas	Capoeira	1B	9	9	48 meses	Recidiva de dor sem sinais de relesão; o paciente seguiu praticando esporte
Masculino	14	10 dias	Basquete	3 (fratura por avulsão)	7	7	24 meses	Soltura com migração de material de síntese com necessidade de revisão: sem intercorrências após a revisão
Masculino	30	8 semanas	Luta ( *mixed martial arts,* MMA, em inglês)	3	9	9	4 meses	Não houve
Masculino	47	6 semanas	Ginásticas olímpica e artística	2C	9	Não disponível	1 mês	Não houve

A idade média foi de 37(±13,2) anos, com variação de 14 a 53 anos. Quanto ao sexo, 9 pacientes (69,2%) eram do sexo masculino. Ao todo, 7 pacientes (53,8%) apresentaram avulsão tendínea completa com retração maior do que de 2 cm (lesão de tipo 3).

A classificação utilizada foi a de Forlizzi et al. 10 (2022), que dividiram as lesões em tipos 1A, 1B, 2C, 2S e 3 (Anexo 1). Para avaliar o desempenho esportivo, foi utilizado o escore de Tegner (Anexo 2), uma escala de avaliação funcional amplamente utilizada para medir o nível de atividade física e esportiva de pacientes, especialmente na ortopedia e na fisioterapia. Ele foi desenvolvido por Tegner e Lysholm 11 em 1985 como complemento à escala de Lysholm, e avalia a capacidade de retorno às atividades esportivas e laborais após lesões no joelho, mas hoje é aplicado em outras articulações. O escore varia de 0 a 10, sendo que 0 indica incapacidade para trabalhar ou praticar esportes devido ao problema articular, e 10, participação em esportes competitivos de alto impacto (como futebol profissional e rúgbi).

### Técnica



**Vídeo 1** Teste após a inserção de âncora metálica.


**Vídeo 2** Sutura do tendão.


**Vídeo 3** Atividade de salto durante a reabilitação.


**Vídeo 4** Fortalecimento dos extensores do quadril na reabilitação.


O paciente é posicionado em decúbito ventral com coxins de proteção ao tórax e com flexão de cerca de 45° dos joelhos (para aproximar o coto e relaxar o nervo ciático), sob raquianestesia e sedação. A incisão acompanha a prega glútea com extensão de 5 a 7 cm, a depender do volume muscular do paciente. Após a abertura da pele e do tecido subcutâneo, é identificada a fáscia do glúteo máximo, que deve ser dissecada evitando-se a lesão do nervo cutâneo femoral posterior. Após a abertura da fáscia e a proteção do nervo, o coto tendíneo deve ser identificado, com grande cautela no seu manejo em virtude da proximidade do nervo ciático. Após a identificação do tendão, é realizada neurólise digital do ciático, e o coto é isolado para posterior reparo. A tuberosidade isquiática deve ser escarificada para propiciar melhor cicatrização do tendão. São utilizadas rotineiramente 3 âncoras metálicas de 5,5 mm, para mimetizar o *footprint* original do tendão (1 âncora para o semimembranoso e 2 para o tendão conjunto), sendo feita sutura contínua e ancorada pelo método de Krackow.

Nos casos com grande retração (> 5 cm após o paciente estar posicionado) e tempo de lesão superior a 3 semanas, uma segunda incisão transversal pode ser realizada sobre a projeção do coto tendíneo, da forma a realizar a liberação da fibrose e facilitar o deslizamento do tendão até a região proximal, para minimizar risco de lesão do nervo ciático.

**Fig. 1:**
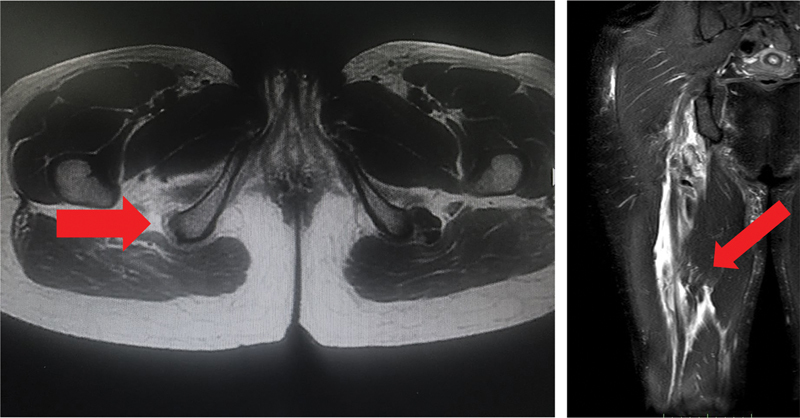
corte axial evidenciando túberisquiático sem a presença do tendão conjunto e do semimembranoso; corte coronal evidencia grande quantidade de líquido e retraç ão do coto tendíneo

O fechamento da(s) incisão(ões) é feito por planos, como é habitual para outros procedimentos. O pós-operatório deve respeitar a proteção da sutura, e deve-se utilizar protocolo de carga parcial com muletas por 2 semanas e restrição para movimento combinado de flexão do quadril com extensão do joelho. A reabilitação, após a quarta semana, pode ser acelerada, buscando retorno aos esportes a partir da décima segunda semana. As etapas da cirurgia estão ilustradas nas Figuras 1 a 9 e nos Vídeos 1 2 3 a 4 .

**Fig. 2:**
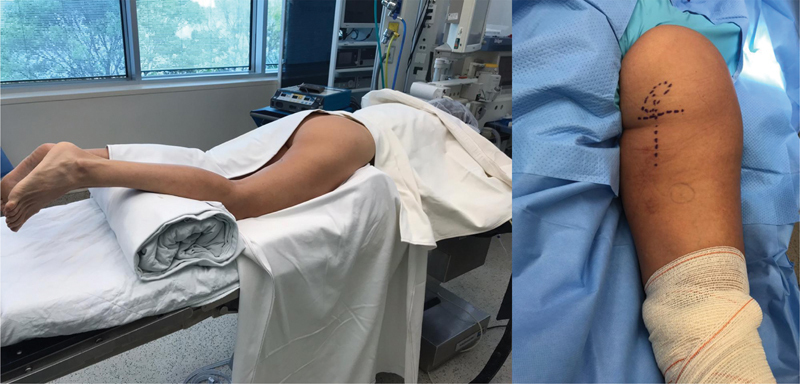
posicionamento para a cirurgia -paciente em decúbito ventral com coxim sob os joelhos para reduzir tensão no coto tendíneo e no nervo ciático; planejamento da incisão transversa na prega glútea e projeç ão longitudinal da retraç ão do coto

**Fig. 3:**
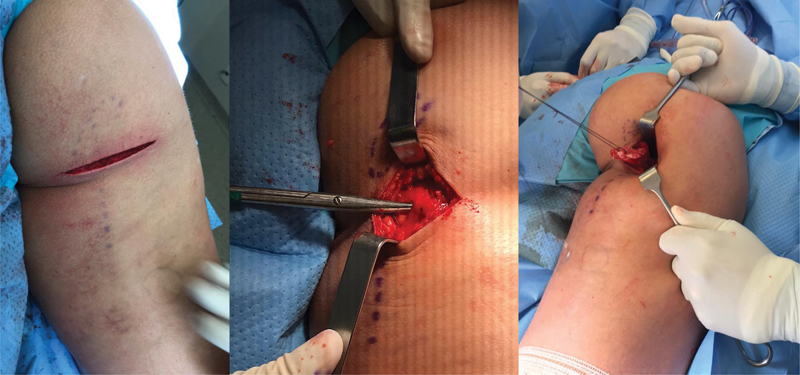
Incisão da pele e subcutâneo; dissecç ão do nervo cutâneo femoral posterior junto à fáscia do glúteo máximo; identificaç ão e reparo do coto tendíneo.

**Fig. 4:**
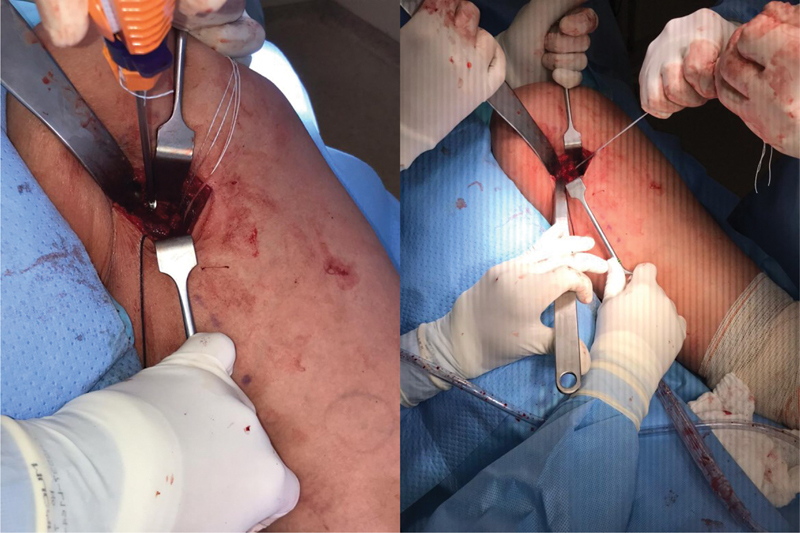
Inserç ão das âncoras metálicas de 5.5 mm no túberisquiático; utilizadas 3 âncoras no footprint (2 para o tendão conjunto e 1 para o semimembranoso)

**Fig. 5:**
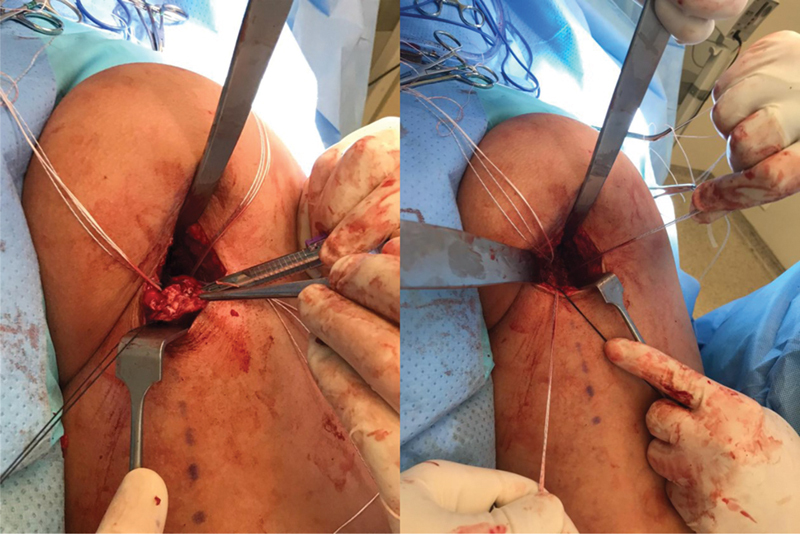
sutura do tendão com pontos contínuos ancorados e reforço com pontos simples.

**Fig. 6:**
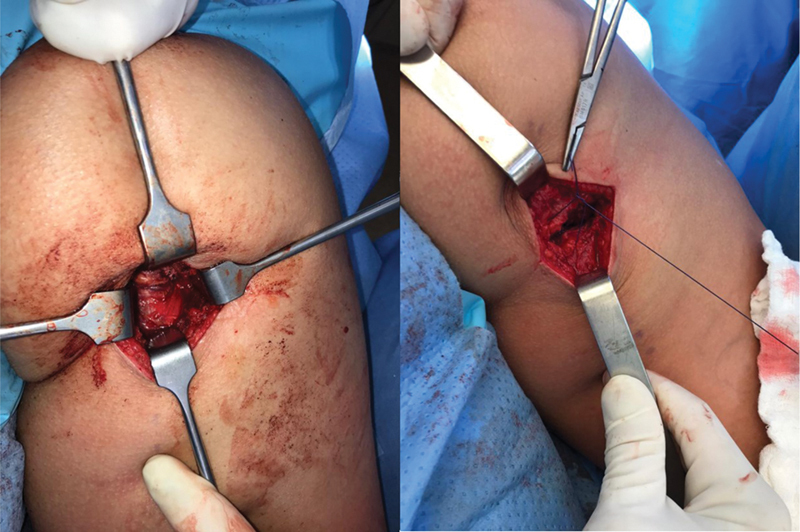
coto tendíneo reinserido no footprint; fechamento da fáscia para evitar aderências.

**Fig. 7:**
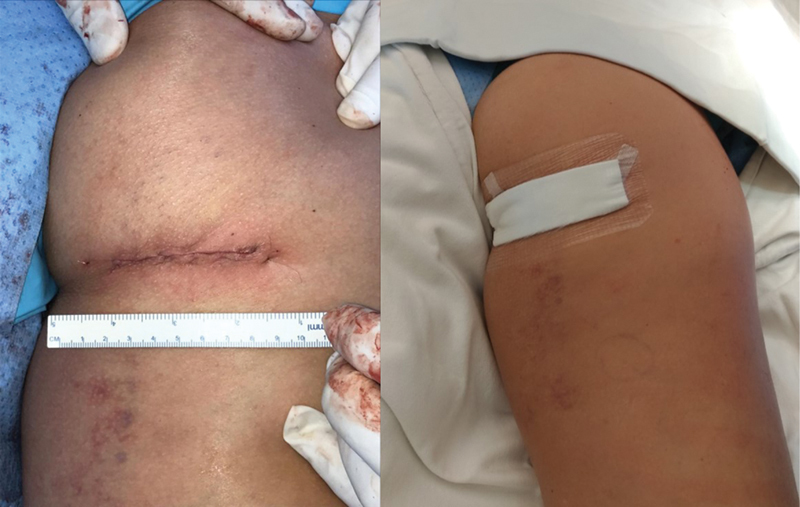
aspecto cosmético da sutura; utilizado curativo impermeável para evitar contaminaç ão secundária

**Fig. 8:**
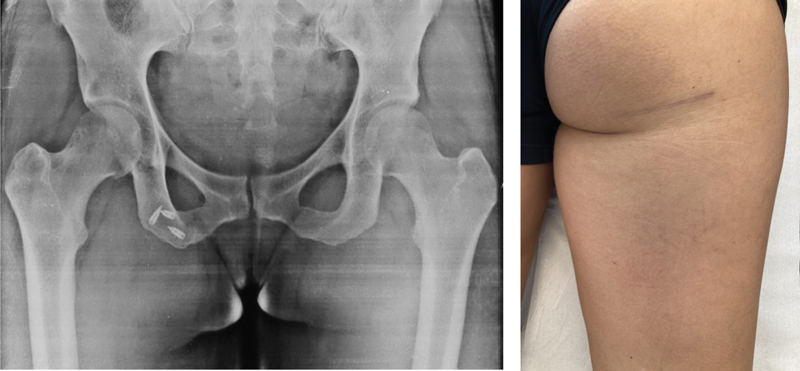
aspecto radiográfico final com as âncoras no footprint do tendão; cicatriz aos 3 meses de pós-operatório

**Fig. 9:**
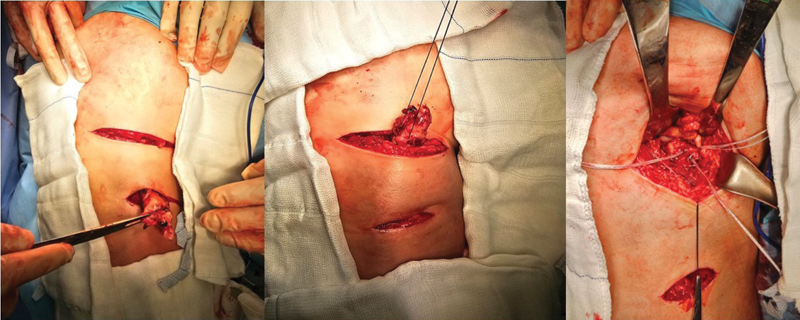
Lesão com 12 semanas e 11 centímetros de retraç ão distal; realizada técnica com dupla incisão transversa.

## Comentários Finais

Utilizamos a técnica apresentada aqui desde 2019, período no qual realizamos o reparo cirúrgico em 13 pacientes. A técnica se mostra reprodutível, com bons resultados clínicos, sem casos de rerruptura até o presente momento, com pontuação pós-operatória no escore de Tegner semelhante à pré-operatória. Para tal, utilizamos o teste de Shapiro-Wilk, com o qual verificamos que os dados não apresentavam distribuição normal; assim, foi aplicado o teste não paramétrico do Wilcoxon para amostras pareadas: média da pontuação pré-operatória no escore de Tegner: 7,1 ± 1,97; média pós-operatória: 7,0 ± 1.83 ( *p* = 0,72), o que evidenciou boa capacidade de recuperação esportiva nos pacientes avaliados.

A técnica cirúrgica habitual para pacientes com retraç ões acima de 5,0 cm é com o uso da incisão longitudinal, uma via de acesso ampla, com maior risco potencial de deiscência de sutura. A técnica transversa apresentada, com uma ou 2 incisões, é factível e uma alternativa para o tratamento cirúrgico das lesões proximais dos isquiotibiais.

As indicações principais foram avulsão tendínea completa ou avulsão do tendão conjunto com retração acima de 2 centímetros, em pacientes ativos fisicamente e com idade menor do que 65 anos.
